# Characterization and Quantification by LC-MS/MS of the Chemical Components of the Heating Products of the Flavonoids Extract in Pollen Typhae for Transformation Rule Exploration

**DOI:** 10.3390/molecules201018352

**Published:** 2015-10-08

**Authors:** Yeqing Chen, Hongli Yu, Hao Wu, Yaozong Pan, Kuilong Wang, Yangping Jin, Chengchao Zhang

**Affiliations:** 1 School of Pharmacy, Nanjing University of Chinese Medicine, Nanjing 210023, China; E-Mails: cyqzs88@163.com (Y.C.); yuhongli76@126.com (H.Y.); pan_yaozong@126.com (Y.P.); wjwkl@126.com (K.W.); yangpingctt@126.com (Y.J.); didiaodexiaojuren@163.com (C.Z.); 2 Jiangsu Key Laboratory of Chinese Medicine Processing, Nanjing University of Chinese Medicine, Nanjing 210023, China; 3 Engineering Center of State Ministry of Education for Standardization of Chinese Medicine Processing, Nanjing 210023, China

**Keywords:** chemical transformation, flavonoids, heating products, liquid chromatography-tandem mass spectroscopy, Pollen Typhae

## Abstract

The Traditional Chinese Medicine herbs Pollen Typhae and Pollen Typhae Carbonisatus have been used as a hemostatic medicine promoting blood clotting for thousands of years. In this study, a reliable, highly sensitive method based on LC-MS/MS has been developed for differentiation of the heating products of total flavonoids in Pollen Typhae (FPT-N). Twenty three peaks were detected and 18 peaks have been structurally identified by comparing retention times, high resolution mass spectrometry data, and fragment ions with those of the reference substances and/or literature data. Additionally, 15 compounds have been quantified by multiple reaction monitoring in the negative ionization mode. It was found that the contents of the characterized compounds differed greatly from each other in FPT-N samples. Among them, the content of huaicarbon B significantly increased at first, while it decreased after heating for 25 min, which could be considered as the characteristic component for distinguishing FPT-N. The present study provided an approach to rapidly distinguish the differences of FPT-N samples. In addition, the actively summarized characteristic fragmentation might help deducing the structure of unknown flavonols compounds. Furthermore, transformation rules of flavonoids during the heating process in carbonisatus development could contribute to hemostatic therapeutic component exploration.

## 1. Introduction

Pollen Typhae (PT), namely Puhuang, is a well-known traditional Chinese medicine used in China, Japan and other Asian countries for more than two thousand years [1]. Having both a hemostatic and anti-thrombotic action, it is widely used in the clinic to treat hemorrhagic diseases, especially for the bleeding caused by obstruction due to congealed blood, such as in menorrhagia caused by trauma, hysteromyoma or heavy bleeding due to retained placenta [1,2]. Flavonoid glycosides are responsible for the herb’s multifaceted pharmacological bioactivities [3,4,5].

Carbonisatus is a process by putting Chinese medicines into a heating container to burn them to blacken or brown the surface, and even form a brown or pale brown interior. In the case of Pollen Typhae its product was widely used in the clinic to treat bleeding diseases [6]. Pollen Typhae Carbonisatus (PTC), which is the legal processed product of PT [1], has obviously enhanced anti-hemorrhagic activity, as suggested by pharmacological and clinical studies [7,8,9]. It was reported that total flavones were the main active components of PTC [10,11]. Comparative analysis between PT and PTC found that the changes in the flavonoid components were obvious [12], with a decreased content in total flavonoid and flavonoid glycosides [13], while content of flavonoid aglycones was greatly increased [12,14]. No studies however have revealed the active components that account for the enhanced anti-hemorrhagic activity.

In this paper, the hypothesis that the hemostatic therapeutic effect could be attributed to transformation of flavonoids in Pollen Typhae (FPT) during the heating process in carbonisatus development was proposed. Thus, it is necessary to characterize and quantify the simulated processing (heating) products of total flavonoids in PT (FPT-N).

The initial analytical work was based on HPLC with UV or diode array detection (DAD) [15,16]. However, their low selectivity and sensitivity cause a disadvantage in the analysis, which has led to the development of methods based on liquid chromatography-tandem mass spectroscopy (LC-MS/MS) [17,18]. According to the literature, LC-PDA-MS and high performance capillary electrophoresis (HPCE) have been developed to determine flavonoids in PT, but these assays mainly focused on only a few components [19,20]. Therefore, it is worthwhile to develop a new sensitive method for characterization and quantitation of the components. Recently, full-scanning high resolution mass analyzers, mainly represented by the time-of-flight (TOF) mass analyzers, have offered new challenges [21]. High resolution and accurate mass MS was used to quantify components with excellent accuracy and high reproducibility [22]. LC-MS/MS instruments operating in Multiple Reaction Monitoring (MRM) are widely used for targeted quantitation in triple quadrupole and hybrid triple quadrupole linear ion trap (QTRAP) systems due to their well-known selectivity and sensitivity [23], especially for the reliable separation of isobaric compounds. Thus, enabling reliable analysis of even very complex matrices represents the key benefit [24].

The aim of this study was to develop and validate a single analytical method employing LC-MS/MS for simultaneously characterization and quantification of FPT-N samples to reveal the preliminary transformation rules of flavonoids in PT during the heating process for carbonisatus development. To the best of our knowledge, no other published method covers such a number of flavonoid analytes in FPT-N samples. Additionally, the transformation rules of flavonoids in PT under heating are reported for the first time.

## 2. Results and Discussion

### 2.1. Sample Preparation Optimization

To obtain efficient dissolution of FPT-N, 30%, 60%, 100% (*v*/*v*) methanol were explored as the extract solvent. It was found that the dissolution efficiency by 60% methanol was higher than with the other two solvents, therefore 60% methanol was chosen for dissolution. Because of the known beneficial effects (higher efficiency, shorter extraction time, less solvent usage) [25], ultrasound-assisted dissolution was considered. Furthermore, dissolution repetitions (1–3 times) and dissolution time (15, 30 and 60 min) were also optimized using ultrasonication. The results showed that ultrasound dissolution twice with 60% methanol for 30 min each was comparatively simple and complete for FPT-N dissolution.

### 2.2. Characterization of Detectable Components in FPT-N

**Figure 1:**
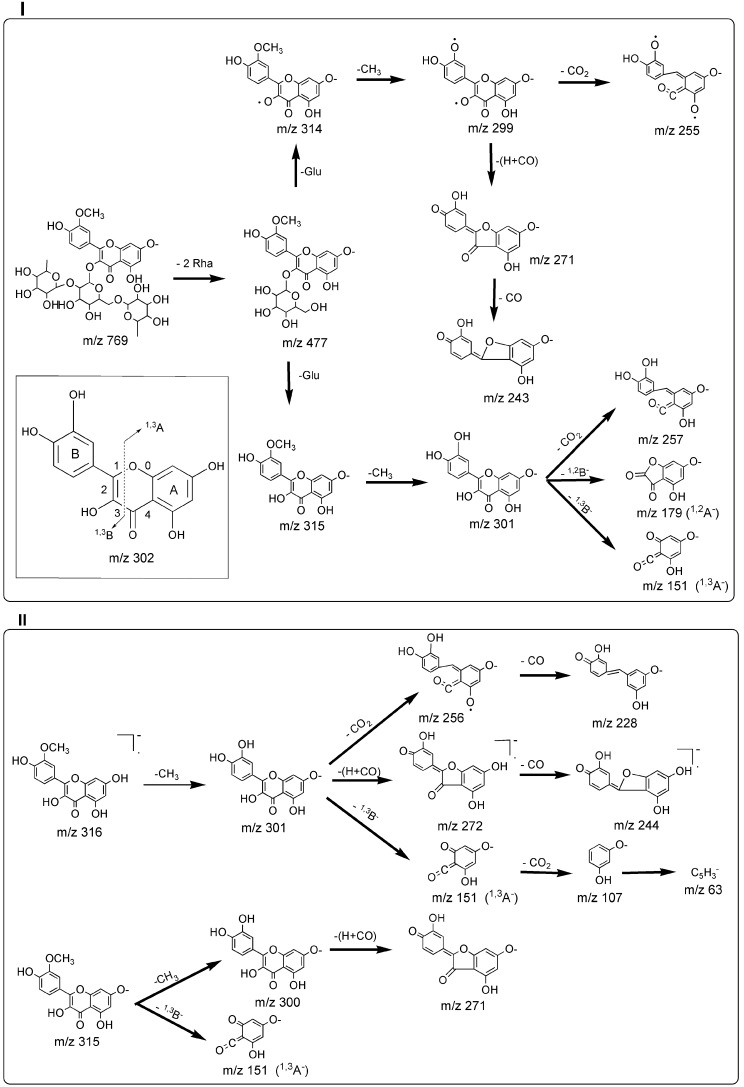
Fragmentation pathways of flavonol-3-*O*-glycoside (typhaneoside, **I**) and its aglycone (isorhamnetin, **II**) proposed on the basis of ESI-MS^n^ spectra in negative mode.

A total of 18 of 23 detected chromatographic peaks were identified in FPT-N. The result is listed in Table 1. Thirteen compounds were identified by the accurate mass information and retention times of targeted reference substances. The structural characterization of the other five compounds was based on the basis of the accurate mass, the registered mass spectra fragmentation patterns and literature data. By summarizing the fragmentation patterns of these reference markers, it can be concluded that simple regular cleavages of consecutive glycosidic bonds in the [M − H]^−^ ion under negative ion mode on the product ions containing the aglycone molecule is observed in the mass spectra of flavonol-3-*O*-glycosides. Thus, flavonol-3-*O*-glycosides were deduced by the successive losses of aglycone fragment ions *m*/*z* 315 (314), *m*/*z* 300 (299), *m*/*z* 285 (284), *m*/*z* 271, *m*/*z* 257 (256, 255), *m*/*z* 243, *m*/*z* 151 (isorhamnetin) or *m*/*z* 301 (300), *m*/*z* 273 (271), *m*/*z* 255, *m*/*z* 243, *m*/*z* 179, *m*/*z* 151, *m*/*z* 121 (quercetin) or *m*/*z* 285 (284), *m*/*z* 255, *m*/*z* 227, *m*/*z* 179, *m*/*z* 151 (kaempferol) from [M − H]^−^ with rhamnose and glucose. Examples of flavonol-3-*O*-glycoside (typhaneoside) and its aglycone (isorhamnetin) are presented in Figure 1.

**Table 1:** Characteristics of 23 peaks from FPT-N by HPLC-MS/MS in negative mode.

Peak No.	t_R_/min	[M − H]^−^ (Error(ppm))	Fragments	Formula	Identification
1	11.08	755.2126(1.0)	300.028, 271.024	C_33_H_40_O_20_	Quercetin-3-*O*-(2^G^-α-l-rhamnosyl)-rutinoside
2	17.10	609.1489(0.5)	300.028, 271.025, 255.029, 178.997	C_27_H_30_O_16_	Quercetin-3-*O*-neohesperidoside
3	18.50	739.2189(−0.3)	575.143, 473.115, 284.033, 255.029, 227.033	C_33_H_40_O_19_	Kaempferol-3-*O*-(2^G^-α-l-rhamnosyl)-rutinoside
4	20.40	769.2280(0.7)	314.043, 299.019, 271.024, 178.998	C_34_H_42_O_20_	Isorhamnetin-3-*O*-2G-rhamnosylrutinoside
5	24.57	609.1520(1.3)	300.028, 271.025, 255.030, 151.002	C_27_H_30_O_16_	Rutin
6	26.62	593.1558(2.8)	473.112, 447.097, 429.083, 327.052, 284.032, 255.029, 227.034, 178.998	C_27_H_30_O_15_	Kaempferol-3-*O*-neohesperidoside
7	27.60	623.1656(3.1)	459.093, 314.042, 299.018, 285.039, 271.023, 257.044, 243.028	C_28_H_32_O_16_	Isorhamnetin-3-*O*-neohesperidoside
8	30.62	261.1301(0.2)	187.098, 169.086, 125.098, 97.068	C_12_H_22_O_6_	Unknown
9	31.10	593.1582(−1.4)	285.041, 255.031, 227.035, 151.003	C_27_H_30_O_15_	Kaempferol-3-*O*-rutinoside
10	32.87	623.1655(2.3)	315.051, 300.027, 271.024, 255.029	C_28_H_32_O_16_	Isorhamnetin-3-*O*-rutinoside
11	33.50	447.0957(1.3)	284.033, 255.030, 227.035,	C_21_H_20_O_11_	Kaempferol-3-*O*-glucoside
12	34.04	767.2160(3.2)	705.213, 665.181, 623.168, 314.045, 299.019, 271.024, 178.996	C_34_H_40_O_20_	Isorhamnetin-3-*O*-rutinoside-7-*O*-rhamnoside
13	34.80	187.0972(−0.4)	125.098, 97.067	C_9_H_16_O_4_	Unknown
14	35.52	477.1072(1.9)	314.043, 299.020, 285.041, 271.025, 257.045, 243.030, 151.003	C_22_H_22_O_12_	Isorhamnetin-3-*O*-glucoside
15	45.85	201.1136(2.5)	183.103, 139.113, 57.039	C_10_H_18_O_4_	Unknown
16	47.12	301.0367(−2.0)	273.042, 243.028, 178.998, 151.004, 121.030	C_15_H_10_O_7_	Quercetin
17	48.09	429.0848(2.1)	411.073, 355.073, 313.035, 301.036, 279.051, 205.013, 163.003, 151.003, 121.029	C_21_H_18_O_10_	Unknown
18	49.49	411.0743(0.6)	369.062, 327.051, 313.036, 202.999, 177.019, 149.024	C_21_H_16_O_9_	Huaicarbon A
19	54.45	271.0633(0.8)	187.039, 151.004, 119.051	C_15_H_12_O_5_	Naringenin
20	54.80	411.0742(1.1)	383.078, 327.051, 261.040, 177.019, 163.003, 121.029	C_21_H_16_O_9_	Huaicarbon B
21	57.02	215.1301(2.7)	197.119, 153.129, 125.098	C_11_H_20_O_4_	Unknown
22	59.00	285.0415(1.0)	239.035, 187.039, 143.050	C_15_H_10_O_6_	Kaempferol
23	62.15	316.0562(1.4)	301.032, 272.029, 256.034, 164.009, 151.004, 107.014	C_16_H_12_O_7_	Isorhamnetin

### 2.3. Comparison of the Chemical Compositions of FPT-N

**Figure 2:**
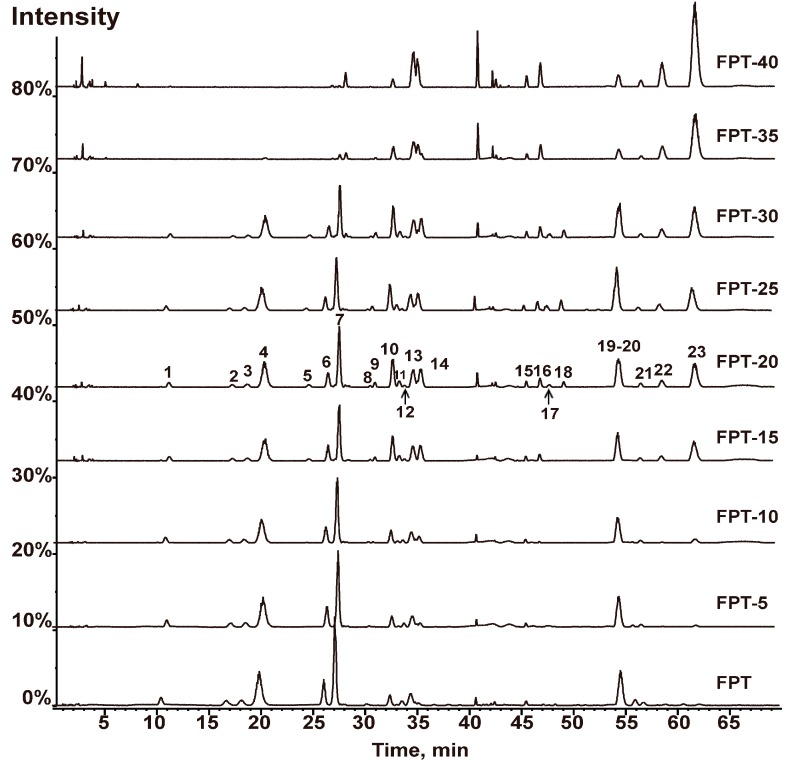
The representative BPC of FPT-N samples based on HPLC-MS/MS. Flavonoids extract in Pollen Typhae (FPT) was heated for 5 min (FPT-5), 10 min (FPT-10), 15 min (FPT-15), 20 (FPT-20), 25 min (FPT-25), 30 min (FPT-30), 35 min (FPT-35), 40 min (FPT-40).

FPT-N samples were comparatively analyzed using HPLC-MS/MS. The representative base peak chromatograms (BPC) of FPT-N are shown in Figure 2. A total of 23 compounds were detected. The peak areas of the 23 compounds are shown in Table 2. The values of the coefficient of variance (C.V.%) of the peak area of compounds were all greater than 24.23%, showing that the peak area of component in different FPT-N samples varied significantly, especially the compounds huaicarbon A and huaicarbon B. The detailed differences between the chemical compositions could also be easily obtained by analyzing the entire FPT-N chromatogram. Compared to FPT, it was interesting to find that the peak heights of the compounds quercetin-3-*O*-(2G-α-L-rhamnosyl)-rutinoside, quercetin-3-*O*-neo-hesperidoside, kaempferol-3-*O*-(2G-α-L-rhamnosyl)-rutinoside, typhaneoside, kaempferol-3-*O*-neo-hesperidoside, isorhamnetin-3-*O*-neohesperidoside, isorhamnetin-3-*O*-rutinoside-7-*O*-rhamnoside and naringenin decreased with extended heating time, but the heights of the compounds kaempferol-3-*O*-rutinoside, isorhamnetin-3-*O*-rutinoside, kaempferol-3-*O*-glucoside and isorhamnetin-3-*O*-glucoside increased first during heating from 0 min to 15 min but then decreased as the heating time was extended. Furthermore, it could be detected that some resultant substances (compounds huaicarbon A and huaicarbon B) were generated observable after heating for 15 min and disappeared after heating for 35 min, these two components in FPT-20, FPT-25 and FPT-30 have been reported to show significant hemostatic effects [6].

**Table 2:** Relative peak area of each peak of FPT-N.

Num.	t_R_/min	Average Peak Area of Every Peak (*n* = 3)									
		FPT	FPT-5	FPT-10	FPT-15	FPT-20	FPT-25	FPT-30	FPT-35	FPT-40	C.V. (%)
1	11.08	338,487	248,945	190,893	105,507	96,305	93,937	69,277	4310	1591	82.66
2	17.10	231,095	144,044	118,210	65,441	57,995	54,765	40,293	0	0	88.50
3	18.50	250,582	157,283	121,377	69,560	65,107	64,139	47,261	3610	1205	86.06
4	20.40	1,444,606	901,553	726,186	427,207	418,008	398,437	319,037	27,993	10,310	81.68
5	24.57	0	0	0	55,020	60,998	54,765	49,918	15,128	7140	95.32
6	26.62	1,042,571	667,434	527,981	325,984	257,649	250,932	183,495	0	0	87.53
7	27.60	3,456,078	2,339,062	1,897,589	1,110,021	1,065,516	968,690	832,223	50,427	34,210	79.36
8	30.62	75,447	50,503	45,368	37,703	37,258	36,274	32,183	15,608	15,728	44.59
9	31.10	0	0	0	0	50,085	56,039	55,660	30,675	13,107	104.88
10	32.87	0	0	0	350,970	240,830	380,980	408,210	157,953	73,290	90.10
11	33.50	38,427	47,841	75,699	120,125	114,629	117,944	103,895	22,196	72,97	57.92
12	34.04	201,798	143,419	96,909	54,688	42,018	40,041	29,285	1525	0	94.14
13	34.80	494,581	361,347	338,906	303,863	307,321	280,237	264,417	214,084	260,209	24.23
14	35.52	116,612	147,941	215,245	331,932	320,628	317,606	285,552	76,463	27,049	53.61
15	45.85	190,865	132,543	114,595	101,895	107,104	98,996	95,422	73,178	89,037	29.75
16	47.12	17,240	25,420	54,195	142,218	159,424	159,116	164,163	171,018	179,377	52.71
17	48.09	0	0	0	0	12,037	48,028	30,523	0	0	138.22
18	49.49	0	0	0	0	40,803	70,827	48,284	0	0	156.31
19	54.45	1,374,905	962,889	745,617	547,685	474,347	416,039	414,197	131,722	97,802	66.36
20	54.80	0	0	0	87,950	445,439	792,825	517,389	92,696	0	136.94
21	57.02	136,603	96,849	88,325	75,154	75,391	71,495	67,002	48,944	58,374	30.34
22	59.00	18,101	18,640	35,045	111,241	125,427	119,013	136,296	159,644	182,316	53.23
23	62.15	61,589	69,451	123,176	405,860	400,656	410,033	438,180	545,758	588,749	59.55

Numbers used for abbreviations (FPT-5, 10, 15, ..., 40) stands for the heating period.

There are some other resultant compounds such as quercetin, kaempferol, isorhamnetin and peak 17 that were generated by heating for 15 min. All the results above suggested that these FPT-N samples showed significant differences due to the 23 different characteristic peaks. Based on all the above results, we can speculate that the *O*-triglycosylated 3-*O*-substituted isorhamnetin (quercetin) is transformed into *O*-di-/*O*-monoglycosylated 3-*O*-substituted isorhamnetin (quercetin) or directly into the corresponding flavonol aglycone isorhamnetin (quercetin) by removal of the sugar moiety or into some other newly generated hemostatic components (huaicarbon A and huaicarbon B) upon heating. However, the structures of the compounds are destroyed, until they completely disappear, after extended heating times.

### 2.4. Method Validation

To evaluate the linearity, calibration standards of six concentration levels of each analyte were prepared and analyzed. The calibration curve was constructed by plotting the peak area *vs.* the concentrations. As shown in Table 3, all 13 target compounds showed good linear regression within the test range. The linear regression analysis results showed that the coefficient estimation of the standard curve was >0.9987. The data showed excellent reproducibility. The LOD were estimated to be 0.15–3.20 ng/mL. The LOQs were estimated to be 0.52–5.66 ng/mL. The results of repeatability, stability and precision for the determination of the analytes are shown in Table 4, which indicating all the relative standard deviation (RSD) values were within 5.0%. The recovery values of the 13 compounds as shown in Table 4 were within the range of 92.03%–103.98%. The above validation data showed that MRM was sensitive for quantitation of the analytes.

### 2.5. Quantification Analysis of the 15 Components in FPT-N Samples

The developed method was applied to quantitatively analyze the 15 ingredients in FPT-N. The quantitative results are summarized in Table 5. The contents determination of qercetin-3-*O*-neohesperidoside and kempferol-3-*O*-neohesperidoside were respectively based on rutin and kaempferol-3-rutinoside with the same precursor/product ions in MRM mode and on the complete chromatographic separation between each other. As shown in Table 6, contents of quercetin-3-*O*-neohesperidoside, typhaneoside, kaempferol-3-*O*-neohesperidoside, isorhamnetin-3-*O*-neo-hesperidoside and naringenin decreased with heating time extending; the content of rutin, kaempferol-3-*O*-rutinoside, isorhamnetin-3-*O*-rutinoside, kaempferol-3-*O*-glucoside, isorhamnetin-3-*O*-glucoside, huaicarbon A and huaicarbon B firstly increased and then decreased after heating for 25 min; on the contrary, contents of quercetin, kaempferol and isorhamnetin increased continuously with heating time. The results indicated that contents of the 15 compounds differed greatly from each other in FPT-N samples, which were consistent with the above results summarized in Section 2.3.

## 3. Experimental Section

### 3.1. Materials and Reagents

Pollen Typhae (10 kg) was collected from Jiangsu Province, China. It was identified by Chungen Wang (College of Pharmacy, Nanjing University of Chinese Medicine) as belonging to the dried pollen of *Typha orientalis* Presl. LC-MS grade methanol, acetonitrile and MS grade formic acid (98%) were purchased from Merck (Darmstadt, Germany). Deionized water was purified by a Milli-Q system (Millipore, Bedford, MA, USA). Analytical reference standards of typhaneoside, rutin, isorhamnetin-3-*O*-neohesperidoside, kaempferol-3-*O*-rutinoside, isorhamnetin-3-*O*-rutinoside, kaempferol-3-*O*-glucoside, isorhamnetin-3-*O*-glucoside, quercetin, huaicarbon A, naringenin, huaicarbon B, kaempferol and isorhamnetin with 98% purity were purchased from the National Institutes for Food and Drug Control (Beijing, China). Stock solutions (about 1.0 mg/mL) were prepared in methanol. Diluted mix standards were prepared with acetonitrile/water (18/82, *v*/*v*). For the validation purposes, working standard solutions mixture at about 1.0 μg/mL was prepared in volumetric flask from stock solutions of particular analytical standards.

**Table 3:** Validation data including the regression equations, correlation coefficient (r), linear ranges, limit of detection (LOQ) and lower limit of quantitation (LOD) for the determination of the analytes.

Analyte	Regression Equation	r	Linear Range (μg/mL)	LOQ (ng/mL)	LOD (ng/mL)
Typhaneoside	y = 5271.12x − 1.1114 × 10^5^	0.9991	12.86–205.75	1.05	0.35
Rutin	y = 3079.77x − 28489.25	0.9992	6.87–110.00	2.81	1.02
Isorhamnetin-3-*O*-neohesperidoside	y = 27,033.66x − 1.49 × 10^6^	0.9996	31.30–450.75	1.12	0.22
Kaempferol-3-*O*-rutinoside	y = 916.76x − 13151.76	0.9996	8.76–100.25	2.81	0.82
Isorhamnetin-3-*O*-rutinoside	y = 4215.53x − 5.66 × 10^4^	0.9992	15.63–250.00	3.27	1.28
Kaempferol-3-*O*-glucoside	y = 4369.04x − 6.26 × 10^4^	0.9994	8.44–135.00	4.30	2.06
Isorhamnetin-3-*O*-glucoside	y = 18,288.43x − 2.17 × 10^5^	0.9990	7.20–115.25	0.72	0.18
Quercetin	y = 1179.82x − 3.09 × 10^4^	0.9991	6.25–100.00	3.75	1.42
Huaicarbon A	y = 3029.48x − 20866.52	0.9990	5.34–85.45	5.36	2.45
Naringenin	y = 8688.43x − 7.31 × 10^4^	0.9994	6.57–105.13	0.52	0.15
Huaicarbon B	y = 898.48x − 5371.09	0.9992	4.69–75.05	0.48	0.20
Kaempferol	y = 1078.54x − 3.61 × 10^4^	0.9989	6.59–105.50	4.32	2.52
Isorhamnetin	y = 5467.97x − 6.13 × 10^4^	0.9987	6.98–111.75	5.66	3.20

**Table 4:** Validation data including repeatability, stability, precision and recovery for the determination of the analytes.

Analyte	RSD (%, *n* = 6)				Accuracy	
	Repeatability	Stability	Precision			
			Intra-day	Inter-day	Recovery (%, *n* = 3)	RSD (%)
Typhaneoside	1.75	0.83	1.46	0.62	98.20	3.8
Rutin	2.02	3.84	2.01	4.02	100.49	4.99
Isorhamnetin-3-*O*-neohesperidoside	1.64	2.85	2.02	2.41	93.82	4.20
Kaempferol-3-*O*-rutinoside	3.67	1.72	2.70	3.05	100.50	4.02
Isorhamnetin-3-*O*-glucoside	2.88	2.85	3.51	0.72	103.98	4.20
Quercetin	3.02	5.73	4.80	4.53	94.28	4.01
Huaicarbon A	4.87	4.42	2.08	1.83	93.92	3.21
Naringenin	4.35	1.83	0.88	1.72	100.35	2.84
Huaicarbon B	3.95	3.07	2.80	3.05	100.31	4.98
Kaempferol	4.07	3.92	4.50	4.21	95.29	3.82
Isorhamnetin	3.55	3.16	3.48	3.07	92.03	4.77

**Table 5:** Contents of the 15 analytes in samples of FPT-N (mg/mg).

Analyte	FPT	FPT-5	FPT-10	FPT-15	FPT-20	FPT-25	FPT-30	FPT-35	FPT-40
Quercetin-3-*O*-neohesperidoside	0.0153	0.0123	0.0095	0.0079	0.0063	0.0052	0.0040	N/A	N/A
Typhaneoside	0.1939	0.1575	0.1304	0.1246	0.0840	0.0682	0.0402	0.0223	0.0152
Rutin	N/A	N/A	N/A	0.008	0.012	0.010	0.006	N/A	N/A
Kaempferol-3-*O*-neohesperidoside	0.0971	0.0702	0.0601	0.0508	0.0442	0.0295	0.0202	0.0084	N/A
Isorhamnetin-3-*O*-neohesperidoside	0.3351	0.2808	0.2403	0.2008	0.1700	0.0955	0.0672	0.0204	0.0052
Kaempferol-3-*O*-rutinoside	N/A	N/A	N/A	0.0095	0.0129	0.0184	0.0122	N/A	N/A
Isorhamnetin-3-*O*-rutinoside	0.0404	0.0428	0.0501	0.0552	0.0606	0.0600	0.0502	0.0404	0.0204
Kaempferol-3-*O*-glucoside	N/A	N/A	0.0071	0.0090	0.0108	0.0116	0.0082	0.0060	0.0046
Isorhamnetin-3-*O*-glucoside	0.0086	0.0185	0.0262	0.0354	0.0680	0.0452	0.0372	0.0244	0.0138
Quercetin	0.0036	0.0039	0.0046	0.0050	0.0056	0.0062	0.0078	0.0092	0.0103
Huaicarbon A	N/A	N/A	N/A	0.0032	0.0045	0.0059	0.0040	N/A	N/A
Naringenin	0.1076	0.1003	0.0950	0.0838	0.0720	0.0584	0.0485	0.0363	0.0304
Huaicarbon B	N/A	N/A	N/A	0.0282	0.0375	0.0484	0.0360	0.0205	N/A
Kaempferol	0.0038	0.0045	0.0058	0.0066	0.0074	0.0082	0.0089	0.0095	0.0195
Isorhamnetin	0.0074	0.0098	0.0106	0.0258	0.0342	0.0444	0.0489	0.0595	0.0626

Numbers used for abbreviations (FPT-5, 10, 15, ..., 40) stands for the heating period. N/A: Not applicable.

**Table 6:** LC-MS/MS characteristics of quantified compounds in negative ion mode. The measured precursor ion, product ions, the declustering potencial (DP), collision energy (CE), as well as the cell exit potential (CEP) are given.

Analyte	t_R_/min	Precursor Ion (*m*/*z*)	Product Ion (*m*/*z*)	DP (V)	CE (V)	CEP (V)
Typhaneoside	3.34	769.077	314.000	−30	−52	−29
Rutin	3.74	608.921	299.900	−175	−48	−25
Isorhamnetin-3-*O*-neohesperidoside	4.27	623.089	313.900	−175	−42	−19
Kaempferol-3-*O*-rutinoside	5.11	593.066	284.500	−160	−46	−29
Isorhamnetin-3-*O*-rutinoside	5.45	623.017	315.000	−195	−44	−17
Kaempferol-3-*O*-glucoside	5.64	447.009	284.000	−145	−36	−33
Isorhamnetin-3-*O*-glucoside	6.00	476.818	314.000	−140	−36	−19
Quercetin	8.80	300.904	150.900	−90	−28	−15
Huaicarbon A	9.31	411.002	326.900	−165	−28	−37
Naringenin	10.69	270.954	151.000	−130	−24	−11
Huaicarbon B	10.83	410.990	327.100	−190	−38	−33
Kaempferol	11.52	284.970	186.900	−160	−36	−11
Isorhamnetin	12.02	315.019	299.900	−90	−30	−37

### 3.2. Sample Preparation

FPT was obtained first to prepare FPT-N. Prior to the extraction, Pollen Typhae were blended with 65% methanol (1:15, *w*/*v*) Section 2.1 says 60% for infiltrating 2 h, and then placed in boiling water bath (temp 95 °C) for 1 h. This step was repeated twice for complete extraction. The extracts were combined, filtered and evaporated with a rotary vacuum evaporator. The concentrated solution was subjected to macroporous resin column chromatography, and eluted with water/ethanol successively in a gradient (10:0 to 0:10). The fractions (eluted with 70% and 80% ethanol) were concentrated to dryness by evaporation to obtain the FPT with 85% purity. FPT-N was prepared using single component heating (TEMP = 200 °C) that simulated the carbonization process. FPT was heated in a muffle furnace for 5 min (FPT-5), 10 min (FPT-10), 15 min (FPT-20), 20 min (FPT-20), 25 (FPT-25), 30 min (FPT-30), 35 min (FPT-35) and 40 min (FPT-40) to obtain FPT-N.

To support efficient dissolution of analytes, and to achieve the best recoveries of analytes from “dry” matrices (FPT-N in our case), ultrasound-assisted dissolution was utilized. fine homogenized FPT-N powder (10 mg) was weighed into screwed glass vial, and 10 mL methanol/water solvent (30/70, 60/40, 100/0, *v*/*v*) was added and left into an ultrasonic bath (Kunshan, Nanjing, China) for 15, 30, 60 min. This step was repeated 1–3 times for complete dissolution. The total extracts were transferred into a 50 mL volumetric flask and the volume was adjusted to the calibration mark by methanol. The supernatant solutions were filtered through a 0.22 μm syringe filter, and then were diluted in the same way as working standard solutions (about 1.0 μg/mL) for LC-MS/MS analysis. One mL of purified extract was removed into a vial prior to injection on LC-MS/MS system.

### 3.3. HPLC-MS/MS Conditions

UFLC XR Ultra high performance liquid chromatograph (Shimadzu, Kyoto, Japan) was used for the separation of target analytes and for the method development. SunFire^TM^ C18 analytical column (100 mm × 2.1 mm, i.d., 3.5 μm particle size; Waters, Dublin, Ireland) coupled with a C18 pre-column (Waters, Milford, CT, USA) maintained at 35 °C was used for quantitation (I) and a Dubhe C18 column (250 mm × 4.6 mm, 5.0 mm; Hanbon, Nanjing, China) maintained was used for characterization (II). To optimize separation and detection conditions, two types of mobile phases were used. The mobile phase was a mixture of 0.1% formic acid–water (A) and acetonitrile (B), with an optimized linear gradient elution as follows: (I) 0–5 min, 15%–20% B; 5–6 min, 20%–28% B; 6–10 min, 28% B; 10–12 min, 28%–35% B; 12–15 min, 35% B. The flow rate was stable at 0.6 mL/min throughout the run. (II) 0–20 min: 15% B; 20–25 min: 18%–20% B; 25–35 min: 20% B; 35–40 min: 20%–30% B; 40–70 min: 30% B. The flow rate was set at 1.0 mL/min with split ratio 1:1.

Mass spectrometry was performed on the Triple TOF^TM^ 5600 (AB SCIEX, Los Angeles, CA, USA) for characterization equipped with electrospray ionization (ESI) source (AB SCIEX). Samples were analyzed in negative ion modes for structural identification and using the following conditions: nebulizer gas (Gas 1) of 55 psi; heater gas (Gas 2) of 55 psi; curtain gas of 35; ion spray voltage of 7 eV; turbo spray temperature (TEM) of 550 °C; declustering potential (DP) of 60 V for MS; declustering potential (DP) of 100 V for MS/MS; collision energy (CE) of 10 for MS; collision energy (CE) of 35 and collision energy spread (CES) of 15 for MS/MS. Compounds were identified by comparing retention time and *m*/*z* values obtained by MS and MS^2^ using PeakView^TM^ software. Peak areas values were obtained using the XIC Manager in PeakView^TM^ software. Extracted ion chromatograms (XICs) were automatically generated for each targeted analyte and compared with a user-defined threshold.

### 3.4. MRM Method Validation

Mass spectrometry was performed on 5500 QTRAP (AB SCIEX) equipped with ESI source operated in negative mode. The parameters were as follows: capillary temperature, 550 °C; curtain gas, 35; ion spray voltage, −4500 V; ion source gas 1 (Gas 1), 55 psi; ion source gas 2 (Gas 2), 55 psi. For each analyte the optimum conditions of MRM were determined in infusion mode are listed in Table 6. Each analyte was directly infused into the electrospray source via a 50 μm i.d. PEEK capillary at a flow rate of 10 μL/min. The quantification was done from the calibration curves obtained in MRM mode by using Multi Quant 2.1.1 software.

The utilized validation concept was based on the guidance [H] GPH5-1 (CFDA, Beijing, China). Linearity, limits of detection (LODs), repeatability, stability, precision and accuracy were carried out under the developed method mentioned above. The “intra-day” and the “inter-day” variances was intended to determine the precision. A recovery experiment of analyte at three spiking levels was performed to investigate the accuracy. A known amount of each of the 15 analytes at three concentrations of 75%, 100% and 125% was added to the corresponding FPT-N powder. Then the resultant samples were extracted and analyzed with the established method in triplets at each level. The estimations of the RSD were based on the ANOVA. The data obtained from the LC-MS/MS were extracted and processed in the same way to permit an unbiased comparison.

## 4. Conclusions

In this paper, a simple, reliable and highly sensitive LC-MS/MS method to characterize and quantify FPT-N samples has been developed and validated. A total of 23 peaks were detected and 18 peaks were identified. In addition, 15 compounds have been quantified. Comparing the chemical compositions in FPT-N, it was found that these FPT-N samples showed significant differences due to 23 different characteristic peaks, especially that due to the hemostatic compound huaicarbon B. Accordingly, in order to guarantee the hemostatic activity of PTC, the heating process should be controlled at 200 °C for 20–30 min. Our data demonstrate that LC-MS/MS may be a promising method for differentiating the original species from their processed products to discover the active compounds. All these results provide some useful references for the application of PT and its processed product PTC in practice.

## Abbreviations

Pollen Typhae (PT); Pollen Typhae Carbonisatus (PTC); Base peak chromatograms (BPC); Liquid chromatography-tandem mass spectroscopy (LC-MS/MS); Flavonoids extract in Pollen Typhae (FPT); heating products of total flavonoids in Pollen Typhae (FPT-N); FPT was heated for 5 min (FPT-5), 10 min (FPT-10), 15 min (FPT-15), 20 (FPT-20), 25 min (FPT-25), 30 min (FPT-30), 35 min (FPT-35), 40 min (FPT-40).
